# Cytogenetics of entelegyne spiders (Arachnida, Araneae) from southern Africa

**DOI:** 10.3897/CompCytogen.v14i1.48667

**Published:** 2020-03-04

**Authors:** František Šťáhlavský, Martin Forman, Pavel Just, Filip Denič, Charles R. Haddad, Vera Opatova

**Affiliations:** 1 Department of Zoology, Charles University, Faculty of Science, Viničná 7, CZ-12844 Praha, Czech Republic; 2 Department of Genetics and Microbiology, Charles University, Faculty of Science, Viničná 5, CZ-12844 Praha, Czech Republic; 3 Department of Zoology and Entomology, University of the Free State, P.O. Box 339, Bloemfontein 9300, South Africa

**Keywords:** Karyotype, sex chromosomes, meiosis, rDNA FISH, NOR, acrocentric, Gnaphosoidea, Araneoidea, Oecobioidea, RTA clade

## Abstract

Spiders represent one of the most studied arachnid orders. They are particularly intriguing from a cytogenetic point of view, due to their complex and dynamic sex chromosome determination systems. Despite intensive research on this group, cytogenetic data from African spiders are still mostly lacking. In this study, we describe the karyotypes of 38 species of spiders belonging to 16 entelegyne families from South Africa and Namibia. In the majority of analysed families, the observed chromosome numbers and morphology (mainly acrocentric) did not deviate from the family-level cytogenetic characteristics based on material from other continents: Tetragnathidae (2n♂ = 24), Ctenidae and Oxyopidae (2n♂ = 28), Sparassidae (2n♂ = 42), Gnaphosidae, Trachelidae and Trochanteriidae (2n♂ = 22), and Salticidae (2n♂ = 28). On the other hand, we identified interspecific variability within Hersiliidae (2n♂ = 33 and 35), Oecobiidae (2n♂ = 19 and 25), Selenopidae (2n♂ = 26 and 29) and Theridiidae (2n♂ = 21 and 22). We examined the karyotypes of Ammoxenidae and Gallieniellidae for the first time. Their diploid counts (2n♂ = 22) correspond to the superfamily Gnaphosoidea and support their placement in this lineage. On the other hand, the karyotypes of Prodidominae (2n♂ = 28 and 29) contrast with all other Gnaphosoidea. Similarly, the unusually high diploid number in *Borboropactus* sp. (2n♂ = 28) within the otherwise cytogenetically uniform family Thomisidae (mainly 2n♂ = 21–24) supports molecular data suggesting a basal position of the genus in the family. The implementation of FISH methods for visualisation of rDNA clusters facilitated the detection of complex dynamics of numbers of these loci. We identified up to five loci of the 18S rDNA clusters in our samples. Three different sex chromosome systems (X0, X_1_X_2_0 and X_1_X_2_X_3_0) were also detected among the studied taxa.

## Introduction

With nearly 50000 species, spiders represent the second largest order of arachnids (World Spider Catalog 2019). The order consists of three main lineages: Mesothelae, Mygalomorphae and Araneomorphae (e.g. Garrison et al. 2016); the last one, with 96 described families, is one of the most diverse groups of arachnids. Araneomorphae (so-called modern spiders) were traditionally divided into Haplogynae and Entelegynae, based on the differences in complexity of their copulatory organs (e.g. Coddington et al. 2004). However, recent phylogenomic studies have disputed Haplogynae monophyly (Bond et al. 2014, Garrison et al. 2016). The monophyly of Entelegynae is well-supported, and the group comprises approximately 80% of extant spider species. Our knowledge about spider cytogenetics also mirrors the diversification disparity among the spider groups. Entelegynae, being the most speciose spider taxon, also represents the most frequently analysed group (86% of all 843 analysed spiders) (Araujo et al. 2019).

Based on their cytogenetic characteristics, spiders can be assigned into several groups displaying different dynamics of karyotype evolution. Their assignment into these groups correlates with the parcelling of spider diversity into the main evolutionary lineages. The basal groups Mesothelae (2n♂ = 80 or 96) (Suzuki 1954) and Mygalomorphae (2n♂ = 14–128, average 61) (Král et al. 2013) typically show higher numbers of chromosomes, a condition regarded as ancestral for spiders (Suzuki 1954). Concerning Araneomorphae, the haplogyne superfamily Dysderoidea represents the most cytogenetically distinct group. It is characterized by lower diploid numbers (2n♂ = 5–40, average 13) and holokinetic chromosomes. This chromosome type is unique in spiders, and evolved only once in this order (e.g. Král et al. 2006, 2019). The rest of haplogyne spiders typically possess a low number of biarmed chromosomes (2n♂ = 9–33, average 21) (Král et al. 2006), with the exception of the presumably polyploid family Caponiidae (2n♂ = 55–152) (Král et al. 2019).

Compared to haplogyne and mygalomorph spiders, the karyotypes of entelegynes are considerably less diversified. Their respective interfamilial diploid number (2n) ranges are low (2n♂ = 10–52, average 27), and the majority of species possess exclusively acrocentric chromosomes (Araujo et al. 2019). The ancestral condition in entelegynes is hypothesised to be 40 acrocentric autosomes (Král et al. 2006), while a reduction in 2n is likely the leading evolutionary trend (Suzuki 1954). An increasing chromosome number in Entelegynae karyotype evolution is theoretically regarded as problematic, since simple fission of an acrocentric chromosome leads to the formation of an acentric fragment. The fragment is unable to attach to a spindle microtubule and thus cannot be transferred into the daughter cell (e.g. Schubert and Lysak 2011).

A prominent feature of spider karyotypes is the presence of unusual sex chromosomes. The majority of species display the X_1_X_2_0 system (male: X_1_X_2_, female: X_1_X_1_X_2_X_2_), which is considered to be an ancestral state in spiders (Suzuki 1954). This system is otherwise rare in animals (White 1976). Interestingly, X_1_X_2_0 evolved into a variety of sex chromosome systems, resulting in an enormous diversity of male heterogamy modes. Namely, systems with up to 13 differentiated X chromosomes in mygalomorphs (Král et al. 2013), X_1_X_2_Y systems in several Haplogynae families (Král et al. 2006), and various neo-sex chromosome systems, reported from three Entelegynae families: Salticidae (Maddison and Leduc-Robert 2013), Agelenidae (Král 2007) and Sparassidae (Sharp and Rowell 2007). In entelegynes, besides the proposed ancestral X_1_X_2_0 and neo-sex chromosome systems, X0 and X_1_X_2_X_3_0 systems are often present (Araujo et al. 2012, Kořínková and Král 2013). Rather exceptionally, some members of three Entelegynae families (Corinnidae, Sparassidae and Tetragnathidae) display an X_1_X_2_X_3_X_4_0 system (Data and Chatterjee 1983, 1988, Araujo et al. 2012). To further add to the complexity of systems of differentiated sex chromosomes in spiders, Král et al. (2013) proposed the existence of additive XY pair(s), with a weak level of differentiation. However, such structures are not distinguishable by conventional karyotype examination in entelegynes.

Due to the conservative aspects of karyotype features in entelegynes, our knowledge of chromosomal evolution in this group could be broadened by the implementation of molecular cytogenetic approaches. Because of the limited number of banding techniques available for invertebrates, the fluorescence *in situ* hybridization (FISH) for visualisation of nucleolus organizer regions (NORs) is a convenient choice in terms of methodology. The NORs are composed of clusters of genes coding most of the rRNA, namely major rDNA loci (18S, 5.8S and 28S rRNA genes). The application of FISH in spider chromosome studies is scarce (Vítková et al. 2005, Zhao et al. 2010, Suzuki and Kubota 2011). Major rDNA clusters were successfully identified via FISH in five species of entelegynes (Forman et al. 2013, Rincão et al. 2017) and one mygalomorph (Král et al. 2013).

Southern Africa includes three of the 36 global biodiversity hotspots (Mittermeier et al. 2011, Noss et al. 2015), with many groups displaying a typical Gondwanan distribution (e.g. Beron 2018). Despite the importance of this geographical region and the relatively good knowledge of cytogenetics in other arachnid groups, e.g. scorpions (e.g. Šťáhlavský et al. 2018a), harvestmen (e.g. Svojanovská et al. 2016, Šťáhlavský et al. 2018b) and pseudoscorpions (e.g. Šťáhlavský et al. 2006, 2012), our knowledge about spiders here is limited. A few attempts have been made to elucidate the karyotype diversity of mygalomorphs and haplogynes from this region (Král et al. 2006, 2013, 2019). However, despite the enormous diversity of entelegynes, and our comparatively good knowledge about their cytogenetics worldwide, there is a significant lack of karyotype data from sub-Saharan Africa (Araujo et al. 2019). So far, only the social spider species *Stegodyphus dumicola* Pocock, 1898 (Eresidae) (2n♂ = 26) has been subjected to cytogenetic analyses (Avilés et al. 1999).

In this study, we analysed the karyotypes of 38 species representing 16 entelegyne families (Araneoidea, Oecobioidea and RTA clade groups) from South Africa and Namibia, to gain knowledge about entelegyne cytogenetics from this biogeographical region. Additionally, we analysed major rDNA clusters via FISH in 11 species. Our results also address the status of Prodidominae and the overall composition of Gnaphosoidea, which highlights the utilization of cytogenetic methods as an important tool to bring additional perspectives for the study of entelegyne taxonomy and systematics. We use the molecular phylogenetic framework and classification established in Wheeler et al. (2016), which represents the most complete assessment of spider diversity to date, and also includes large number of genera sampled and karyotyped in our study.

## Material and methods

Specimen and locality data of 55 entelegyne samples (38 species) analysed in this study are reported in Table 1. Vouchers were deposited in the National Museum, Bloemfontein, South Africa (NMBA). Our analyses were based exclusively on males, in order to determine the sex chromosome systems based on the analysis of meiosis in the heterogametic sex. Chromosome preparations were obtained by the “plate spreading” method (Traut 1976), adapted for arachnids (Šťáhlavský and Král 2004). During the procedure, gonads are hypotonized in 0.075 M KCl (20 min), fixed in methanol: acetic acid (3:1) solution (20 min), dissociated and spread in a drop of 60% acetic acid on a microscope slide on a hot plate (40–45 °C). The chromosomes were stained in a 5% Giemsa solution in modified Sörensen phosphate buffer (30 min) (Dolejš et al. 2011).

**Table 1.** List of examined species, including summary of the cytogenetic data: 2n of male, chromosome morphology (A = completely acrocentric), sex chromosome system, length ratio of sex chromosomes (N = number of measured nuclei) and number of NOR loci. Locality data (EC – Eastern Cape; FS – Free State; MP – Mpumalanga; NAM – Namibia; NL – KwaZulu-Natal; NP – National Park; WC – Western Cape; ZA – South Africa) and sample size (m mature male, sm submature male). * = unidentifiable specimens.

Family/*species*	2n	Chromosome morphology	Sex chromosome system	X ratio (N)	Number of 18S rDNA loci	Locality	GPS (S/E)	Sample size
**I. Araneoidea**								
** Tetragnathidae **								
*Pachygnatha* sp.	24	A	X_1_X_2_0	1:0.96 (8)	–	ZA-MP: God‘s Window	24.8747, 30.8910	1m
** Theridiidae **								
Argyrodes cf. convivans Lawrence, 1937	21	A	X0	–	4	NL: Tembe	27.0276, 32.4083	1m
*Argyrodes* sp.	21	A	X0	–	–	NL: Ndumo	26.8749, 32.2109	2m
Theridion cf. purcelli O. P.-Cambridge, 1904	22	A	X_1_X_2_0	1:0.80 (6)	–	NL: Pongola Reserve	27.3601, 31.9848	1m
**II. Oecobioidea**								
** Hersiliidae **								
*Hersilia sericea* Pocock, 1898	35	A	X_1_X_2_X_3_0	1:0.92:0.71 (9)	–	NL: Vernon Crookes	30.2749, 30.6092	1m
*Neotama corticola* (Lawrence, 1937)	33	A	X_1_X_2_X_3_0	1:0.89:0.76 (10)	–	ZA-EC: Port St. Johns	31.5977, 29.5346	1m
** Oecobiidae **								
*Oecobius navus* Blackwall, 1859	19	A	X0	–	–	ZA-EC: Hogsback	32.5914, 26.9303	2m
*Oecobius putus* O. P.-Cambridge, 1876	25	A	X_1_X_2_X_3_0	–	–	ZA-FS: Bloemfontein	29.0949, 26.1621	2m
**III. RTA - non-Dionycha**								
** Ctenidae **								
Ctenus cf. pulchriventris (Simon, 1896)	28	A	X_1_X_2_0	1:0.86 (14)	–	ZA-MP: Sudwala Caves	25.3713, 30.6965	2m
** Oxyopidae **								
*Peucetia striata* Karsch, 1878	28	A	X_1_X_2_0	1:0.84 (4)	–	ZA-FS: Bloemfontein	29.0488, 26.2152	1m
** Sparassidae **								
*Olios* sp.	42	A	X_1_X_2_0	1:0.93 (20)	1	NL: Ndumo	26.8749, 32.2109	1sm
Sparassinae sp. cf. *Olios*	42	A	X_1_X_2_0	1:0.93 (20)	4	NAM: south of Etosha	19.6208, 15.8858	1sm
** Thomisidae **								
*Borboropactus* sp.	28	A	X_1_X_2_0	1:0.76 (5)	–	NL: Pietermaritzburg	29.6050, 30.3462	1sm
*Xysticus* sp.	23	A	X0	–	–	NL: Ndumo	26.8749, 32.2109	2sm
**IVa. RTA clade Dionycha - “Prodidomidae Simon, 1884, Prodidominae (*sensu*Azevedo et al. 2018)**”								
*Prodidomus simoni* Dalmas, 1919	29	A	X1X2X30	1:0.94:0.91 (7)	–	NL: Ndumo	26.8855, 32.3124	4m
*Theuma* sp.	28	A	X1X20	1:0.68 (12)	–	ZA-FS: Bloemfontein	29.04876, 6.2152	1sm
**IVb. RTA - Dionycha Part A - (Gnaphosoidea*sensu lato*, (Wheeler et al. 2016))**								
** Ammoxenidae **								
*Ammoxenus amphalodes* Dippenaar & Meyer, 1980	22	A	X_1_X_2_0	?	–	ZA-FS: Bloemfontein	29.0986, 26.1550	1m
*Ammoxenus psammodromus* Simon, 1910	22	A	X_1_X_2_0	1:0.86 (4)	–	ZA-FS: Bloemfontein	29.0986, 26.1550	1m
** Gallieniellidae **								
*Austrachelas natalensis* Lawrence, 1942	22	A?	X_1_X_2_0	1:0.80 (8)	–	NL: Ithala Reserve	27.5426, 31.2824	1m
** Gnaphosidae **								
*Camillina maun* Platnick & Murphy, 1987	22	A	X_1_X_2_0	1:0.90 (4)	–	NL: Cornationweg	27.6946, 31.0609	1m
*Camillina maun* Platnick & Murphy, 1987	22	A	X_1_X_2_0	1:0.92 (11)		NL: Manzengenya	27.2361, 32.7076	2m
*Zelotes fuligineus* (Purcell, 1907)	22	–	X_1_X_2_0	1:0.94 (7)	–	NL: Cornationweg	27.6946, 31.0609	1m
*Zelotes sclateri* Tucker, 1923	22	A	X_1_X_2_0	1:0.82 (10)	2	NL: Ithala Reserve	27.5426, 31.2824	2m
*Zelotes sclateri* Tucker, 1923	22	A	X_1_X_2_0	1:0.88 (10)	–	NL: Ndumo	26.8855, 32.3124	1m
*Zelotes* sp.	22	A	X_1_X_2_0	1:0.85 (8)	–	NL: Ndumo	26.8854, 32.3124	1sm
** Trachelidae **								
*Afroceto plana* Lyle & Haddad, 2010	22	A	X_1_X_2_0	1:0.85 (23)	1	NL: Ndumo	26.8855, 32.3124	2m
** Trochanteriidae **								
*Platyoides walteri* (Karsch, 1887)	22	A	X_1_X_2_0	1:0.93 (10)	3	NL: Royal Natal NP	28.7101, 28.9336	1m, 1sm
**IVc. RTA clade - Dionycha Part B**								
** Cheiracanthiidae **								
*Cheiramiona kirkspriggsi* Lotz, 2015	24	A	X_1_X_2_0	1:0.77 (6)	1	NL: Ithala Reserve	27.5426, 31.2824	1m
** Salticidae **								
*Baryphas ahenus* Simon, 1902	28	A	X_1_X_2_0	1:0.98 (7)	–	NL: Tembe	27.0276, 32.4083	1sm
*Cyrba lineata* Wanless, 1984	28	A	X_1_X_2_0	1:0.95 (16)	–	NL: Ndumo	26.8749, 32.2109	2m
*Holcolaetis zuluensis* Lawrence, 1937	28	–	X_1_X_2_0	1:0.76 (4)	–	NL: Ndumo	26.8855, 32.3124	1m
*Myrmarachne laurentina* Bacelar, 1953	28	–	X_1_X_2_0	1:0.84 (6)	–	NL: Ndumo	26.8855, 32.3124	1m
*Menemerus minshullae* Wesołowska, 1999	28	A	X_1_X_2_0	1:53 (7)	–	NL: Ndumo	26.8749, 32.2109	1m
*Nigorella hirsuta* Wesołowska, 2009	28	A	X_1_X_2_0	1:0.92 (7)	–	ZA-FS: Bloemfontein	29.0483, 26.2112	1m
*Thyene ogdeni* Peckham & Peckham, 1903	28	A	X_1_X_2_0	1:0.89 (10)	–	NL: Tembe	27.0276, 32.4083	1m
*Thyenula haddadi* Wesołowska, Azarkina & Russell-Smith, 2014	28	A	X_1_X_2_0	1:0.90 (16)	2	NL: Royal Natal NP	28.6909, 28.9415	1m
*Thyenula leighi* (Peckham & Peckham, 1903)	28	A	X_1_X_2_0	1:0.84 (10)	–	NL: Ophathe	28.3742, 31.3898	1m
** Selenopidae **								
*Anyphops* sp.*	26	A	X_1_X_2_0	1:0.77 (8)	–	ZA-WC: Mossel Bay	34.1634, 22.1065	1m
*Anyphops* sp.*	26	A	X_1_X_2_0	1:0.75 (9)	4	NL: Ndumo	26.8749, 32.2109	1sm
*Selenops* sp. 1*	26	A	X_1_X_2_0	1:0.78 (19)	4	NL: Pongola Reserve	27.3602, 31.9848	1sm
*Selenops* sp. 1*	26	A	X_1_X_2_0	1:0.76 (34)	–	NL: Ophathe	28.3937, 31.3942	1sm
*Selenops* sp. 2*	29	A	X_1_X_2_X_3_0	1:0.94:0.87 (15)	1	NAM: Omuthiya	18.3770, 16.6005	1 m

Chromosomes were documented with an ORCA-AG monochromatic camera (Hamamatsu) on an Olympus IX81 microscope operated by Cell^R. Standard karyotype characteristics, such as number, relative size, and morphology of the chromosomes, were analysed from photographs using the LEVAN plugin (Sakamoto and Zacaro 2009) for the IMAGEJ 1.47 program (http://imagej.nih.gov/ij/). The 2n was established by analysing at least ten well-spread nuclei for each species. Classification of chromosome morphology follows Levan et al. (1964). The sex chromosome systems of certain species were identified during meiosis of the heterogametic sex, either by segregation or their behaviour in prophase I (see e.g. Král et al. 2011).

Major rDNA clusters were detected by FISH, with the 18S rDNA probe, as described in Forman et al. (2013). Briefly, biotine-labelled probe was hybridized on the chromosomal preparations. Signal was detected by streptavidine-Cy3, followed by one round of signal amplification using biotinylated antistreptavidine and streptavidine-Cy3. The chromosomes were stained with Fluoroshield with DAPI (4',6-diamidino-2-phenylindole) (Sigma-Aldrich) and observed on an Olympus IX81 microscope with an ORCA-ER camera (Hamamatsu). The photographs were pseudocolored (red for Cy3 and blue for DAPI) and superimposed with Cell^R software (Olympus).

## Results and discussion

We obtained cytogenetic data for 38 species of entelegyne spiders belonging to 16 families (Table 1). Except for the cosmopolitan *Oecobius putus* Blackwall, 1859 (Oecobiidae), all taxonomically determined species were analysed for the first time. We also provide the first cytogenetic information for the spider families Ammoxenidae and Gallieniellidae, and the gnaphosid subfamily Prodidominae, which until recently (Azevedo et al. 2018) was considered as an independent family. The 2n of the examined species ranged from 19 to 42. The observed sex chromosome systems were either X0, X_1_X_2_0 or X_1_X_2_X_3_0. The X_1_X_2_0 was the most frequently occurring system (detected in 75% of species). The acrocentric morphology of the whole chromosome complement was detected in 92% of species. Chromosomes of the remaining species were most likely acrocentric as well, but the lack of well-spread plates prevented us from determining the morphology of certain chromosomes. We present our results divided into four main groups: Araneoidea, Oecobioidea, RTA clade (except Dionycha), and Dionycha (subdivided into three parts), according to their placement within Entelegynae phylogeny (Wheeler et al. 2016). Results of FISH are presented and discussed in a separate section, as well as general aspects of Entelegynae karyotype evolution.

### I. Araneoidea

The superfamily Araneoidea comprises more than 15 families of ecribellate orb-weavers (Wheeler et al. 2016, Dimitrov et al. 2017, Fernández et al. 2018). We analysed members of two families of this highly diversified group, which presents the first insights into the cytogenetics of African araneoids.

**Tetragnathidae Menge, 1866**

**Figure 1.**
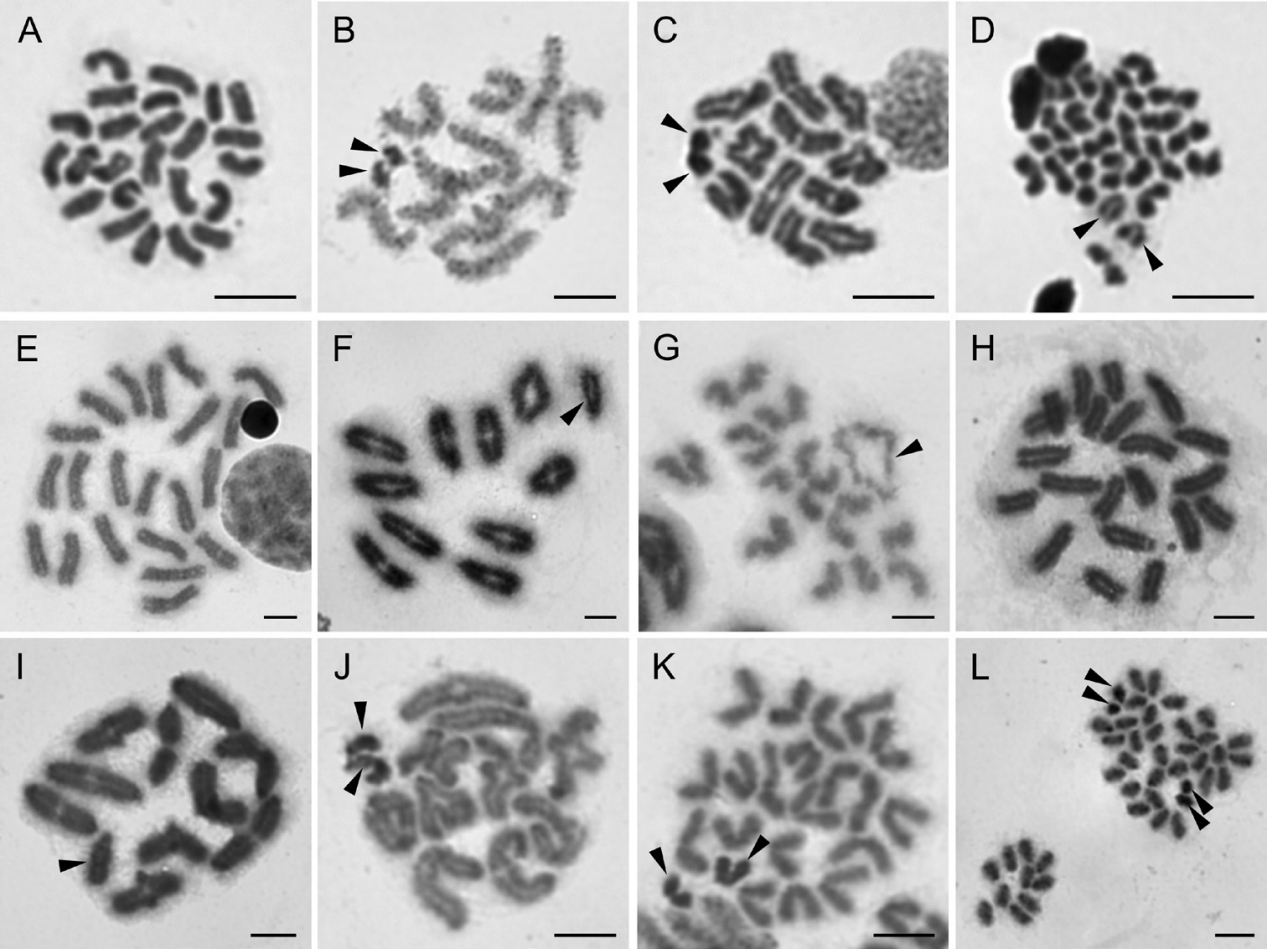
Chromosomes of Tetragnathidae (**A–D**) and Theridiidae (**E–L**). *Pachygnatha* sp. (2n♂ = 24, X_1_X_2_0) **A** mitotic metaphase **B** late pachytene with positively heteropycnotic sex chromosomes **C** diakinesis **D** half of metaphase II, with slightly less condensed X chromosomes. Argyrodes cf. convivans (2n♂ = 21, X0) **E** mitotic metaphase **F** diakinesis with isopycnotic X univalent **G** half of metaphase II with despiralised acrocentric X chromosome. *Argyrodes* sp. (2n♂ = 21, X0) **H** mitotic metaphase **I** diakinesis with isopycnotic X chromosome. Theridion cf. purcelli (2n♂ = 22, X_1_X_2_0) **J** diplotene, X_1_X_2_ associate on the periphery of the plate **K** metaphase II **L** anaphase II. Arrowheads indicate sex chromosomes. Scale bars: 5 μm.

Tetragnathidae is a species-rich family with a cosmopolitan distribution, with 25 species represented in South Africa (Dippenaar-Schoeman et al. 2010, World Spider Catalog 2019). We examined an undetermined species of *Pachygnatha* Sundevall, 1823. We found its karyotype complement displaying 24 chromosomes, gradually decreasing in size (Fig. 1A), with an X_1_X_2_0 sex system and acrocentric morphology in all chromosomes (Fig. 1D). These findings are consistent with karyotypes known from other studied members of the genus (e.g. Gorlov et al. 1995). During the course of prophase I, sex chromosomes exhibited distinctive associations by their centromeres and superspiralization (Fig. 1B, C). Contrary, in metaphase II condensation of the X became weaker (Fig. 1D), as reported in other tetragnathids (Král et al. 2011).

**Theridiidae Sundevall, 1833**

This cosmopolitan and diverse family includes about 57 South African species (Dippenaar-Schoeman et al. 2010, World Spider Catalog 2019). Our dataset included two distinct species of *Argyrodes* Simon, 1864 and Theridion cf. purcelli O. P.-Cambridge, 1904 (Table 1). Both *Argyrodes* displayed 21 acrocentric chromosomes (Fig. 1E, H). We detected one X chromosome, which was positively heteropycnotic from preleptotene until pachytene (not shown), rather less condensed in metaphase II (Fig. 1G), and subsequently slightly positively heteropycnotic in anaphase II. The X chromosome was acrocentric (Fig. 1F, G, I). In contrast, Theridion cf. purcelli showed 2n♂ = 22, with an X_1_X_2_0 sex chromosome system. Its chromosomes were acrocentric and both X displayed positive heteropycnosis from pachytene to anaphase II (Fig. 1J–L). Other members of both genera have often been reported to possess 22 acrocentric chromosomes (Datta and Chatterjee 1983, Srivastava and Shukla 1986, but see latter for *Argyrodes*), which is frequently the case in other theridiids too (Araujo et al. 2019).

### II. Oecobioidea

Alongside the families Uloboridae and Deinopidae, the superfamily Oecobioidea (comprising families Hersiliidae and Oecobiidae) forms the so-called “UDOH grade” (Fernández et al. 2018), which is consistently recovered by molecular data in proximity to the RTA clade (Garrison et al. 2016, Wheeler et al. 2016, Fernández et al. 2018). The superfamily Oecobioidea historically formed part of Eresoidea; however, this grouping was never supported by molecular analyses (Miller et al. 2010, Wheeler et al. 2016, Fernández et al. 2018).

**Hersiliidae Thorell, 1870**

**Figure 2.**
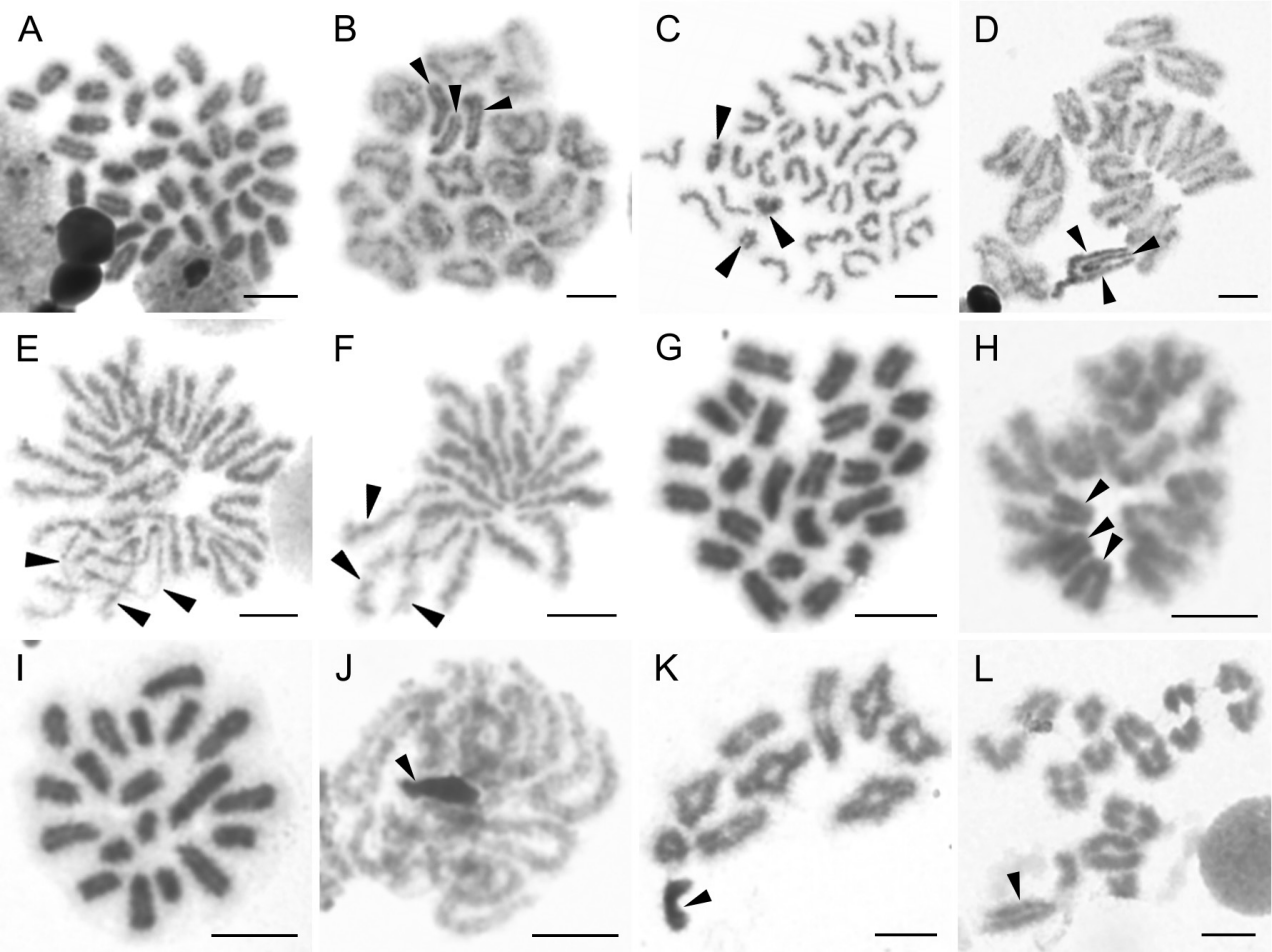
Chromosomes of Hersiliidae (**A–F**) and Oecobiidae (**G–L**). *Hersilia sericea* (2n♂ = 35, X_1_X_2_X_3_0) **A** mitotic metaphase **B** early metaphase I, X_1_X_2_X_3_ shows slightly positive heteropycnosis **C** metaphase II, note positively heteropycnotic X_1_X_2_X_3_. *Neotama corticola* (2n♂ = 33, X_1_X_2_X_3_0) **D** early diakinesis, sex chromosomes **E** half of anaphase I with despiralised sex chromosomes **F** quarter of anaphase II with despiralised sex chromosomes. *Oecobius putus* (2n♂ = 25, X_1_X_2_X_3_0) **G** mitotic metaphase **H** half of anaphase I with positively heteropycnotic sex chromosomes. *O. navus* (2n♂ = 19, X0) **I** mitotic metaphase **J** pachytene with compact X **K** diakinesis X univalent shows positive heteropycnosis **L** metaphase I with already isopycnotic X. Arrowheads indicate sex chromosomes. Scale bars: 5 μm.

Hersiliids are a small family distributed in the tropics and subtropics. Twelve species have been reported from South Africa (Dippenaar-Schoeman et al. 2010, World Spider Catalog 2019). We analysed two of them: *Hersilia sericea* Pocock, 1898 and *Neotama corticola* (Lawrence, 1937). Male diploid counts were 35 and 33, respectively (Fig. 2A–F). Complements of both species were fully acrocentric (Fig. 2C, E, F). Both species exhibited X_1_X_2_X_3_0. Different spiralization of the sex chromosomes, reflected by positive or negative heteropycnosis, was apparent during meiosis (Fig. 2B–F). The karyotype of *H. sericea* differed from its congener *H. savignyi* Lucas, 1836 (Bole-Gowda 1958) by the higher 2n and an additional X chromosome. On the other hand, the same karyotype formula was found in *Hersiliola bayrami* Danişman, Sancak, Erdek & Coşar, 2012 (Kumbıçak et al. 2018). This discrepancy reflects rather higher dynamics in the karyotypes of hersiliids, as seen in *Neotama* Baehr & Baehr, 1993 (this study), as well as in other members of the family (Forman et al. in prep.), and a tendency for convergent 2n reduction in entelegynes (Kořínková and Král 2013).

**Oecobiidae Blackwall, 1862**

The cosmopolitan family Oecobiidae consists of about a hundred species, of which five can be found in South Africa (Dippenaar-Schoeman et al. 2010, World Spider Catalog 2019). Two representatives of the genus *Oecobius* Lucas, 1846 (*O. navus* Blackwall, 1859 and *O. putus* O. Pickard-Cambridge, 1876) were subjects of our investigation. This genus contains small cribellate spiders, including a few synanthropic, cosmopolitan species. In agreement with the previous findings of Mittal (1983), we found the *O. putus* male karyotype to contain an acrocentric set of 25 chromosomes and an X_1_X_2_X_3_0 sex chromosome system (Fig. 2G, H). In contrast, we found the chromosomal complement of *O. navus* to be substantially different from the previous species, comprising 19 acrocentric chromosomes (Fig. 2I). The karyotype constitution of *O. navus* was likely derived by a series of chromosomal fusions. Interestingly, these rearrangements also involved the sex chromosome complement, and resulted in an X0 formation. A fusion-based origin of the *O. navus* karyotype is supported by two features: 1) a low ability of acrocentric chromosomes to be subjected to fissions, and 2) by the length of X in *O. navus*, which was the longest chromosome of the karyotype (Fig. 2K, L). The behaviour of both X_1_X_2_X_3_0 and X0 sex chromosomes in male meiosis included positive heteropycnosis in early prophase (Fig. 2J), which also persisted in the latter phases (Fig. 2K). Karyotype variability of *Oecobius* is unusually high for an entelegyne genus; a male formula of 2n♂ = 22, X_1_X_2_0 is also known from *O. cellariorum* (Dugès, 1836) (Youju et al. 1993).

**RTA clade**

The RTA clade comprises lineages united by the presence of the retrolateral tibial apophysis on the male palps (Griswold et al. 2005). The internal relationships of this group, and also the group itself, did not receive sufficient support in analyses based on traditional Sanger-sequenced loci (Wheeler et al. 2016). In recent phylogenomic analyses, albeit with less exhaustive taxon sampling, the RTA clade was recovered as a monophyletic clade with mostly resolved relationships (Garrison et al. 2016, Fernández et al. 2018). These analyses yielded a similar organization the of RTA clade into several main subclades, but their internal relationships often differed (Garrison et al. 2016, Wheeler et al. 2016, Fernández et al. 2018). Following Wheeler’s et al. (2016) classification, we obtained results from both Dionycha and non-Dionycha clades.

### III. RTA clade non-Dionycha

Here we studied four non-Dionycha lineages, namely three families belonging to the Oval calamistrum clade (Ctenidae, Oxyopidae and Thomisidae) and the family Sparassidae.

**Ctenidae Keyserling, 1877**

**Figure 3.**
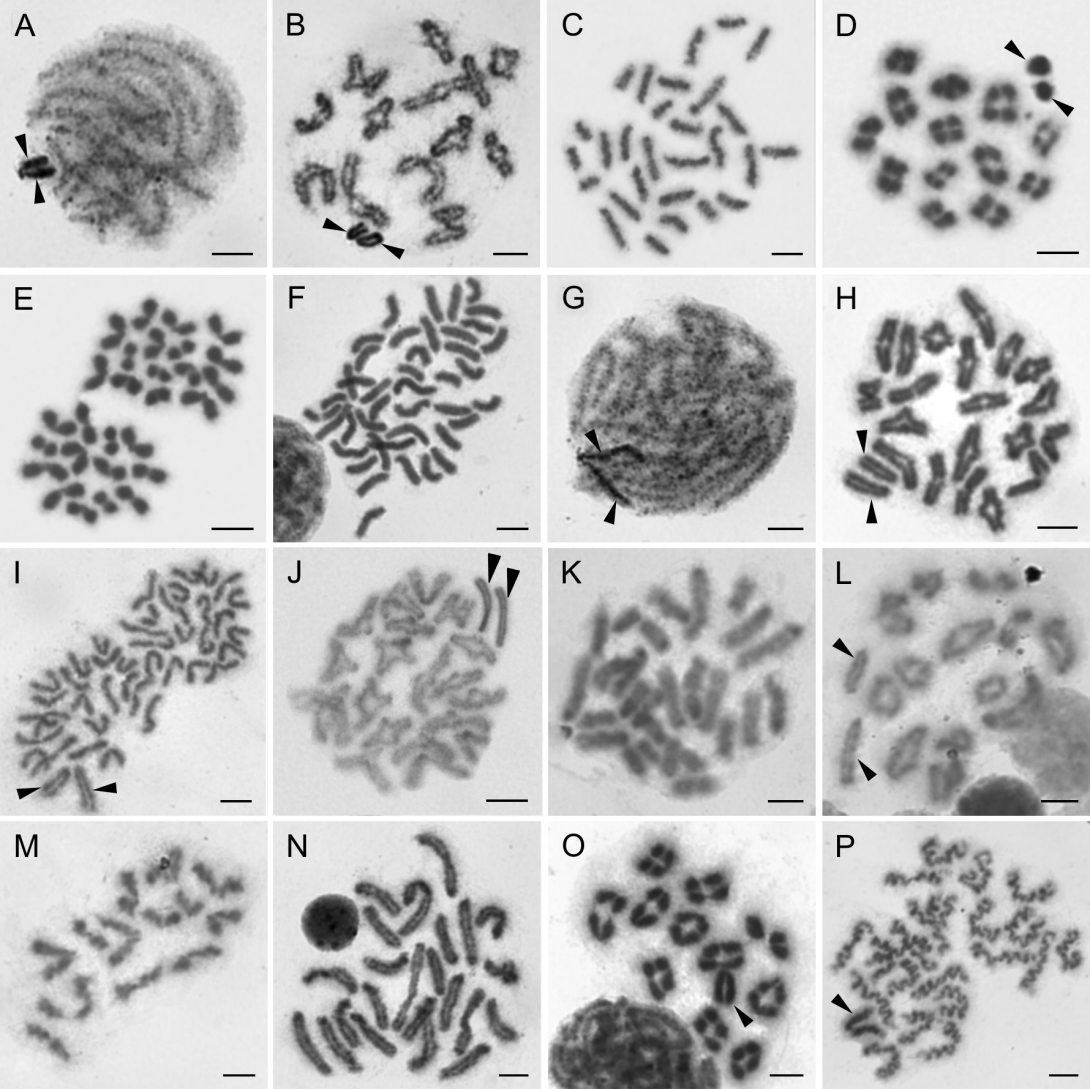
Chromosomes of Ctenidae (**A–B**), Oxyopidae (**C–E**), Sparassidae (**F–J**) and Thomisidae (**K–P**). Ctenus cf. pulchriventris (2n♂ = 28, X_1_X_2_0) **A** pachytene, X_1_X_2_ associate on the periphery of plate **B** diakinesis. *Peucetia striata* (2n♂ = 28, X_1_X_2_0) **C** mitotic metaphase **D** metaphase I **E** metaphase II, sex chromosomes isopycnotic. *Olios* sp. (2n♂ = 42, X_1_X_2_0) **F** mitotic metaphase **G** pachytene **H** late diakinesis **I** metaphase II **J** Diakinesis of Sparassinae sp. cf. *Olios* (2n♂ = 42, X_1_X_2_0). *Borboropactus* sp. (2n♂ = 28, X_1_X_2_0) **K** mitotic metaphase **L** metaphase I **M** half of metaphase II without sex chromosomes. *Xysticus* sp. (2n♂ = 23, X0) **N** mitotic metaphase **O** metaphase I, note early segregation of one bivalent **P** prometaphase II. Arrowheads indicate sex chromosomes. Scale bars: 5 μm.

Wandering spiders are distributed worldwide, except for New Zealand (Jocqué and Dippenaar-Schoeman 2006). They are represented by approximately 520 species, of which seven are known from South Africa (Dippenaar-Schoeman et al. 2010, World Spider Catalog 2019). The family as a whole was not recovered as monophyletic (Wheeler et al. 2016); however, both *Anahita* Karsch, 1879 and *Ctenus* Walckenaer, 1805 belong to the monophyletic “core ctenids”. Since the first report of a ctenid karyotype, of *Anahita fauna* Karsch, 1879 by Chen (1999), our knowledge of the cytogenetics of this family has increased considerably, now comprising data for 11 species, with male karyotypes of 2n♂ = 22, X_1_X_2_0, 2n♂ = 28, X_1_X_2_0, and 2n♂ = 29, X_1_X_2_X_3_0 (Araujo et al. 2014, Kumar et al. 2017, Rincão et al. 2017). Here we report the karyotype of Ctenus cf. pulchriventris (Simon, 1897). We observed 28 chromosomes in the male of this species (Fig. 3B). We were able to confirm acrocentric morphology of all autosomes and both X chromosomes (Fig. 9A). The X chromosomes displayed positive heteropycnosis and parallel associations from pachytene (Fig. 3A) to early metaphase I (Fig. 3B), followed by higher condensation (or late decondensation) in anaphase I (not shown). Both 2n = 28 and X_1_X_2_0 represented the most common constitution in males of Ctenidae, and have been reported so far from eight species, including all examined species of the genus *Ctenus* (Araujo et al. 2019).

**Oxyopidae Thorell, 1870**

Lynx spiders comprise more than 450 species distributed all over the world, of which 41 species have been recorded from South Africa (Dippenaar-Schoeman et al. 2010, World Spider Catalog 2019). In this paper, we analysed the species *Peucetia striata* Karsch, 1878. The male of this species displayed a diploid number of 28 chromosomes (Fig. 3C), with the X_1_X_2_0 sex chromosome system. Sex chromosomes differed slightly in length (the ratio of sex chromosomes - 1:0.86) (Fig. 3D, Table 1). The sex chromosomes showed a different pattern of staining from the autosomes during meiosis I. This positive heteropycnosis was evident from early prophase I (not shown) until metaphase I (Fig. 3D). Later, the sex chromosomes became isopycnotic, and it was not possible to distinguish them from autosomes during metaphase II (Fig. 3E).

Currently, 26 species belonging to five genera have been analysed cytogenetically (Araujo et al. 2019). Our results from *P. striata* from South Africa were similar to the characteristics of Indian (Bole-Gowda 1950, Parida and Sharma 1987, Sharma and Parida 1987) and Turkish (Kumbıçak 2014) representatives of the genus. The main trends in the karyotype evolution of Oxyopidae were the reduction of 2n and change of the sex chromosome system to an X0 type (Stávale et al. 2011). These changes were also observed in two species of *Peucetia* Thorell, 1869 from Brazil (Stávale et al. 2011), and were particularly evident in the genus *Oxyopes* Latreille, 1804 (see Araujo et al. 2019). The centric fusions of the chromosomes in *Oxyopes salticus* Hentz, 1845 resulted in one of the lowest diploid numbers (2n♂ = 11, X0) known among entelegyne spiders (Stávale et al. 2011).

**Sparassidae Bertkau, 1872**

Huntsman spiders, represented by 56 species in South Africa (Dippenaar-Schoeman et al. 2010), are a diverse family predominantly found between 40°N to 40°S latitude, with the exception of the Palearctic genus *Micrommata* Latreille, 1804 (Jocqué and Dippenaar-Schoeman 2006, World Spider Catalog 2019). The family was placed with low support as sister to the Oval calamistrum clade + Dionycha (Wheeler et al. 2016). However, in recent phylogenomic analyses (Fernández et al. 2018) the family was recovered as sister to a clade that would roughly correspond to the “marronoid clade” in Wheeler et al. (2016). We analysed *Olios* sp. from South Africa (Fig. 3F–I) and one penultimate male of an unidentified genus resembling *Olios* Walckenaer, 1837 from Namibia (Fig. 3J). Both males possessed 2n = 42 (Fig. 3F, J), an X_1_X_2_0 sex chromosome system (Fig. 3H, J) and acrocentric morphology of all chromosomes (Fig. 3F, I). Sex chromosomes differed only slightly in their length (the ratio of sex chromosomes – 1:0.93) (Table 1) and showed positive heteropycnosis only during early prophase until pachytene (Fig. 3G). During this phase, they were associated by their centromeric regions (Fig. 3G), and were later located together at the periphery of the nucleus (Fig. 3H–J). The karyotypes of both specimens analysed here show the same characteristics as *O. lamarcki* (Latreille, 1806) from India (Bole-Gowda 1952), whereas *Olios* sp. from Australia possesses an additional sex chromosome (Rowell 1985). Both X_1_X_2_0 and X_1_X_2_X_3_0 systems are common in Sparassidae (see Araujo et al. 2019); exceptions include unique X_1_X_2_X_3_X_4_0 (Datta and Chatterjee 1983) or neo-sex chromosome systems (Sharp and Rowell 2007).

**Thomisidae Sundevall, 1833**

Crab spiders represent a diverse cosmopolitan family, with more than 130 species known from South Africa (Dippenaar-Schoeman et al. 2010, World Spider Catalog 2019). We analysed two unidentified species belonging to the genera *Borboropactus* Simon, 1884 and *Xysticus* C. L. Koch, 1835. The male of *Borboropactus* sp. displayed 28 acrocentric chromosomes (Fig. 3K) and an X_1_X_2_0 system. The sex chromosomes showed conspicuous difference in length (Fig. 3L, Table 1). *Xysticus* sp., on the other hand, displayed 23 acrocentric chromosomes (Fig. 3N), including a single acrocentric X (Fig. 3O, P), which corresponds to the characteristics of the genus reported in the literature (e.g. Hackman 1948, Gorlov et al. 1995, Kumbıçak et al. 2014) and is also typical for most thomisid genera (see Araujo et al. 2019). Presumably, the 2n decreases in Entelegynae karyotype evolution (Suzuki 1954, Kořínková and Král 2013). The higher number of chromosomes detected in *Borboropactus* would thus indicate an ancestral position within Thomisidae, which was further supported by the results of molecular phylogenetic analyses recovering *Borboropactus* at the base of the Thomisidae clade, albeit with low support (Benjamin et al. 2008, Wheeler et al. 2016). However, Wunderlich (2004) doubted the genus’ placement within Thomisidae and established a monogeneric family Borboropactidae (but see Wheeler et al. 2016). The phylogenetic position of the genus, along with the fact that all other thomisids display a lower diploid number, support that 2n♂ = 28, X_1_X_2_0 represents an ancestral condition in this family. Our results thus suggest that the reduction of 2n, accompanied by X chromosome fusions, could play a role in the karyotype evolution of Thomisidae.

### IV. RTA clade Dionycha

Dionycha, the two-clawed spiders, are a diverse group comprising about 17 families, representing a third of known spider species diversity. The group received moderate support in Wheeler et al. (2016), but the internal relationships remain largely unresolved. The exact composition of Dionycha also became a matter of debate recently, due to the conflicting position of Sparassidae (Ramírez 2014, Wheeler et al. 2016, Fernández et al. 2018). The molecular analyses recovered most of the Dionycha diversity placed in three main clades (Wheeler et al. 2016). The first clade comprised Prodidomidae, a family that was traditionally placed within Gnaphosoidea (see below); the second clade, “Dionycha part A”, comprised most of the Gnaphosoidea and few other families; and the third clade, “Dionycha part B”, included corinnids, jumping spiders, miturgids and other families.

Albeit with limited sampling, the phylogenomic analyses recovered the group as monophyletic, with its subdivision into two main clades concordant with the “Dionycha part A” and “Dionycha part B” (Fernández et al. 2018). Based on a morphological analysis of Gnaphosoidea, the family Prodidomidae was transferred to Gnaphosidae, losing its family-level status (Azevedo et al. 2018), whereas the remaining gnaphosoid families remained valid. In this paper, we analysed eight dionychan families from South Africa, including the subfamily Prodidominae (Gnaphosidae). This sampling comprises species representing all of the major Dionycha clades (*sensu*Wheeler et al. 2016), including the first cytogenetic records of Prodidominae, Ammoxenidae and Gallieniellidae.

### IVa. “Prodidomidae Simon, 1884, Prodidominae (*sensu*Azevedo et al. 2018)”

The position of prodidomines remains uncertain. In molecular analyses, they were placed as a sister lineage to all remaining Dionycha (Wheeler et al. 2016). However, based on morphological evidence, the family was recently transferred to Gnaphosidae and established as one of its subfamilies (Azevedo et al. 2018). Prodidomines comprise over 300 species with a tropical and subtropical distribution, of which 26 species are known from South Africa (Dippenaar-Schoeman et al. 2010, World Spider Catalog 2019). In this paper, we report the first chromosomal data for the group.

**Figure 4.**
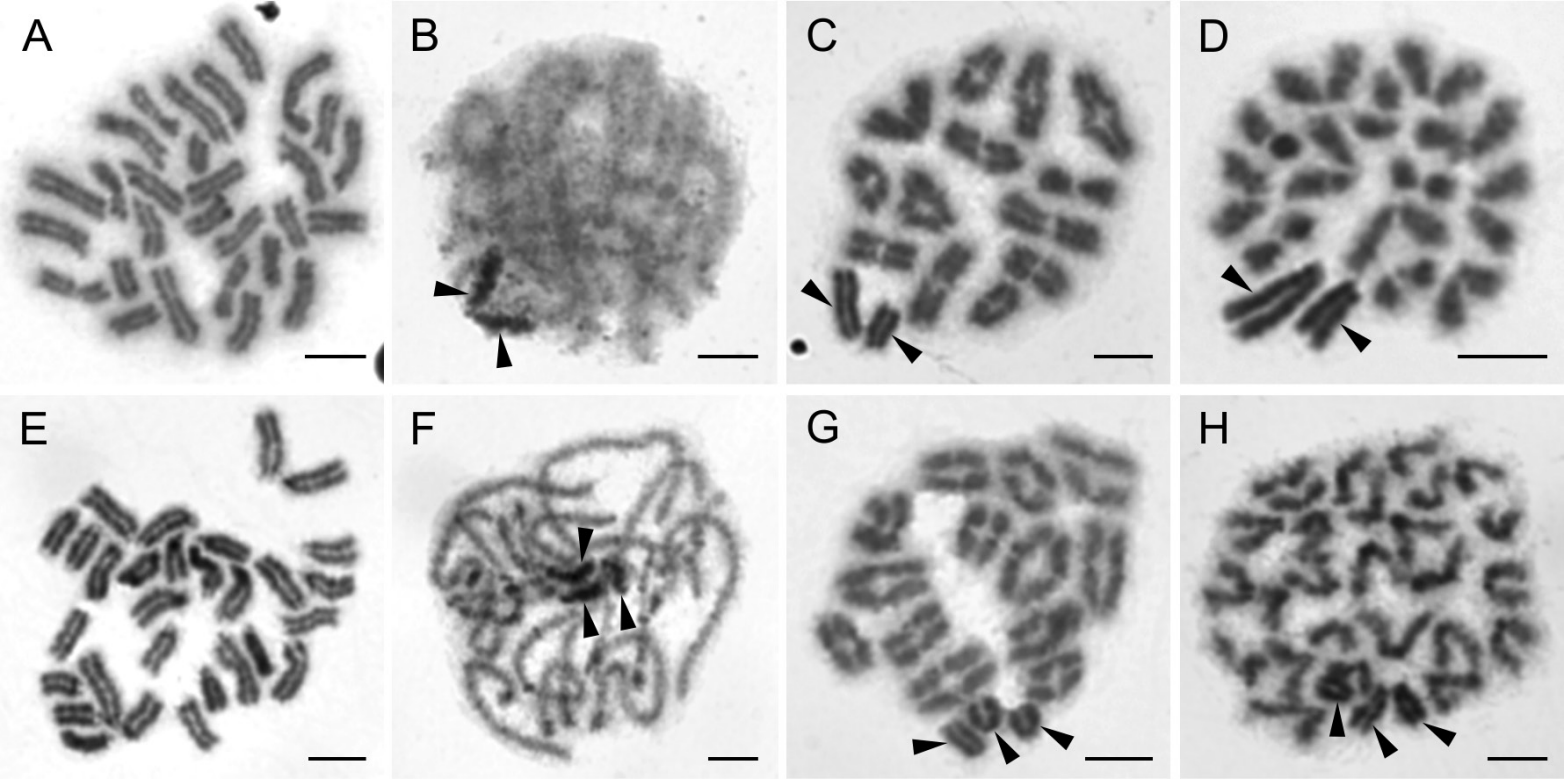
Chromosomes of Prodidominae (Dionycha). *Theuma* sp. (2n♂ = 28, X_1_X_2_0) **A** mitotic metaphase **B** early pachytene with positively heteropycnotic X_1_X_2_**C** diakinesis, note a difference in size of sex chromosomes **D** half of late metaphase II with sex chromosomes. *Prodidomus simoni* (2n♂ = 29, X_1_X_2_X_3_0) **E** mitotic metaphase **F** pachytene note positively heteropycnotic X_1_X_2_X_3_**G** metaphase I **H** early metaphase II, note positively heteropycnotic sex chromosomes. Arrowheads indicate sex chromosomes. Scale bars: 5 μm.

We analysed two species, *Theuma* sp. and *Prodidomus simoni* Dalmas, 1919, representing two formerly recognized prodidomid subfamilies, Theuminae and Prodidominae, respectively (Wheeler et al. 2016). The subadult male of *Theuma* sp. displayed 2n = 28 (Fig. 4A) and an X_1_X_2_0 sex chromosome system. All chromosomes were acrocentric (Fig. 4A, D). The X_1_ and X_2_ sex chromosomes differed considerably in length (the ratio of sex chromosomes – 1:0.68) (Fig. 4C, D, Table 1) and showed positive heteropycnosis during meiosis I (Fig. 4B, C), as well as during meiosis II (Fig. 4D). They were associated by their centromeric regions during pachytene (Fig. 4B), and later in prophase I they remained in close proximity to each other at the periphery of the nucleus (Fig. 4C, D). The males of *P. simoni* also possessed acrocentric chromosomes (Fig. 4H), but displayed a higher chromosome number of 2n = 29 (Fig. 4E), due to a different sex chromosome system. The species possessed an X_1_X_2_X_3_0 system, with the sex chromosomes similar in length (the ratio of sex chromosomes – 1:0.94:0.91, respectively). The course of the heteropycnosis was similar to *Theuma* sp.; the chromosomes were positively heteropycnotic and closely located during the whole meiosis I (Fig. 4F–H). Interestingly, the differences in the length of the sex chromosomes between both species indicated either a fusion of two X chromosomes or fission of X_1_. Despite the data presented here constituting the only information about the chromosomes of prodidomines, they bring a relevant perspective on the placement of the family within Gnaphosidae (see below).

### IVb. RTA clade Dionycha Part A – [Gnaphosoidea*sensu lato* (Wheeler et al. 2016)]

Altogether, we analysed nine species from five families belonging to this clade, and provide the first insights into the karyotypes of Ammoxenidae and Gallieniellidae. Several of the families analysed here, namely Ammoxenidae, Gallieniellidae and Trochanteriidae, were not recovered as monophyletic in previous molecular and morphological phylogenetic analyses, and formed a grade of lineages within the Gnaphosoidea (Wheeler et al. 2016). Therefore, we interpret the obtained results only in the context of Gnaphosoidea.

**Ammoxenidae Simon, 1893**

**Figure 5.**
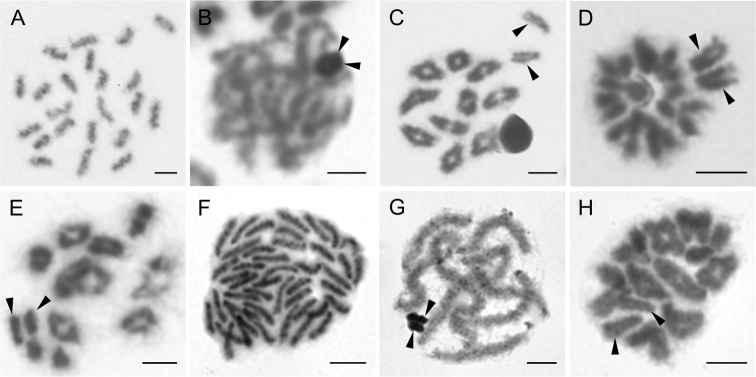
Chromosomes of Ammoxenidae (**A–E**) and Gallieniellidae (**F–H**). *Ammoxenus psammodromus* (2n♂ = 22, X_1_X_2_0) **A** mitotic prometaphase **B** pachytene **C** metaphase I with slightly negatively heteropycnotic X_1_X_2_**D** half of anaphase II including sex chromosomes. *A. amphalodes* (2n♂ = 22, X_1_X_2_0) **E** metaphase I. *Austrachelas natalensis* (2n♂ = 22, X_1_X_2_0) **F** mitotic anaphase, orientation of chromatids suggests acrocentric morphology of chromosomes **G** pachytene X_1_X_2_ shows strong positive heteropycnosis **H** early metaphase I with already isopycnotic sex chromosomes. Arrowheads indicate sex chromosomes. Scale bars: 5 μm.

Ammoxenidae is a small family of termitophagous spiders, currently comprising four genera and 18 species distributed across southern Africa and Australia (World Spider Catalog 2019). We analysed two species of the genus *Ammoxenus* Simon, 1893 (*A. amphalodes* Dippenaar & Meyer, 1980 and *A. psammodromus* Simon, 1910). Males of *A. psammodromus* had 22 acrocentric chromosomes including an X_1_X_2_0 sex chromosome system (Fig. 5A–D). The X chromosomes of this species paired together during pachytene (Fig. 5B) and showed positive heteropycnosis. Subsequently, they became isopycnotic in metaphase I and II (Fig. 5C). The X_1_ and X_2_ differed slightly in length (Table 1). Despite the limited results we obtained for male *A. amphalodes*, we were able to confirm 2n = 22, X_1_X_2_0 (Fig. 5E) and acrocentric chromosomes (Fig. 9B) in this species too.

**Gallieniellidae Millot, 1947**

Recent molecular analyses cast doubts on the monophyly of this family, splitting the group into two lineages with uncertain placement within “Dionycha part A” clade (Wheeler et al. 2016). The family Gallieniellidae has a typical Gondwanan distribution, spanning across the Afrotropical region, Madagascar, Australia and Argentina (Jocqué and Dippenaar-Schoeman 2006). We analysed one species, *Austrachelas natalensis* Lawrence, 1942, which displayed 2n♂ = 22, including an X_1_X_2_0 sex chromosome system. The orientation of the chromosomes in mitotic anaphase suggests an acrocentric morphology (Fig. 5F). The length of the autosomes decreased gradually, and the X_1_ and X_2_ differed slightly in length (the ratio of sex chromosomes – 1:0.80) during diakinesis (Fig. 5H). They showed intensive positive heteropycnosis and parallel associations during pachytene (Fig. 5G), and became isopycnotic in diakinesis and metaphase I (Fig. 5H).

**Gnaphosidae Pocock, 1898**

**Figure 6.**
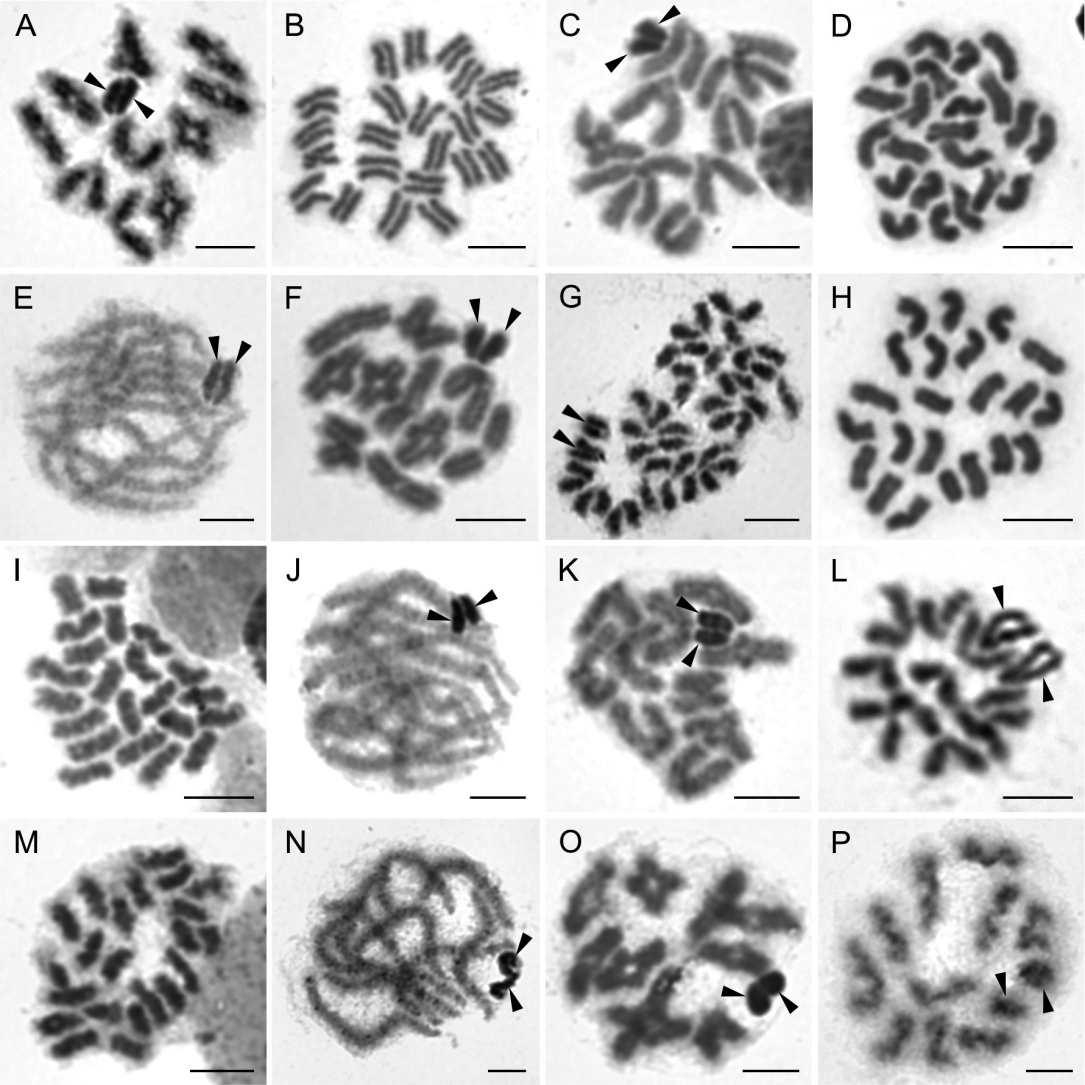
Chromosomes of Gnaphosidae (**A–H**), Trachelidae (**I–L**) and Trochanteriidae (**M–P**) **A** diakinesis of *Camillina maun* (2n♂ = 22, X_1_X_2_0). *Zelotes fuligineus* (2n♂ = 22, X_1_X_2_0) **B** late mitotic metaphase **C** diakinesis, X_1_X_2_ show positive heteropycnosis. *Z. sclateri* (2n♂ = 22, X_1_X_2_0) **D** mitotic metaphase **E** pachytene, sex chromosomes pair in parallel on the periphery of nucleus **F** diakinesis **G** metaphase II, sex chromosomes are nearly isopycnotic **H** mitotic metaphase of *Zelotes* sp. (2n♂ = 22, X_1_X_2_0). *Afroceto plana* (2n♂ = 22, X_1_X_2_0) **I** mitotic metaphase **J** pachytene, X_1_X_2_ show parallel association **K** diakinesis **L** half of metaphase II with sex chromosomes. *Platyoides walteri* (2n♂ = 22, X_1_X_2_0) **M** mitotic metaphase **N** pachytene, sex chromosome associate by their centromeric regions **O** diakinesis, with positively heteropycnotic sex chromosomes **P** half of prometaphase II with sex chromosomes. Arrowheads indicate sex chromosomes. Scale bars: 5 μm.

Gnaphosidae is a diverse family with a cosmopolitan distribution. We analysed four species belonging to the genera *Camillina* Berland, 1919 and *Zelotes* Gistel, 1848, both belonging to the subfamily Zelotinae. The males of all species displayed a diploid number of 22 chromosomes and X_1_X_2_0 sex chromosome system (Fig. 6A–H). The acrocentric morphology of all chromosomes was confirmed in *Camillina maun* Platnick & Murphy, 1987 (Fig. 9C), *Zelotes sclateri* Tucker, 1923 (Fig. 6G), and *Zelotes* sp. (Fig. 9D). During pachytene to late diakinesis, the X_1_ and X_2_ were displaying positive heteropycnosis and pairing in parallel on the periphery of nuclei (Fig. 6A, C, E, F). Despite the enormous species diversity of the family, gnaphosids show extremely conservative karyotype properties, with 2n♂ = 22 and X_1_X_2_0 representing the most common constitution (Araujo et al. 2019).

**Trachelidae Simon, 1897**

Trachelids, recently elevated to family level (Ramírez 2014), include more than 230 species distributed worldwide with exception of Australia (World Spider Catalog 2019). We analysed one South African representative, *Afroceto plana* Lyle & Haddad, 2010. The males of this species had 2n = 22, X_1_X_2_0 (Fig. 6I), with all chromosomes being acrocentric (Fig. 6L). The X_1_ and X_2_ differed slightly in length (the ratio of sex chromosomes – 1:0.85) (Fig. 6J, K, Table 1). Both sex chromosomes displayed intensive positive heteropycnosis and parallel association during pachytene (Fig. 6J). Both heteropycnosis and their location on the periphery of the nucleus persisted during diakinesis (Fig. 6K) and metaphase II (Fig. 6L). The karyotype characteristics, i.e. the 2n, chromosome morphology and the sex chromosome system, fully correspond to the karyotype of *Trachelas japonicus* Bösenberg & Strand, 1906 from Japan (Suzuki 1952). *Trachelas* sp. from India, representing the only remaining analysed trachelid, possesses 2n♂ = 24, X_1_X_2_0 (Datta and Chatterjee 1983).

**Trochanteriidae Karsch, 1879**

Trochanteriidae is another gnaphosoid family with a mainly Gondwanan distribution, but also extending to East Asia. In South Africa, the family is represented by nine species of the genus *Platyoides* O. Pickard-Cambridge, 1891 (Dippenaar-Schoeman et al. 2010, World Spider Catalog 2019), of which we analysed one species. *Platyoides walteri* (Karsch, 1887) displayed 2n♂ = 22, X_1_X_2_0 (Fig. 6M, O), with all of the chromosomes being acrocentric (Fig. 6M, P) and decreasing gradually in length. X_2_ was smaller than X_1_ and probably represented the smallest chromosome of the complement. Sex chromosomes were positively heteropycnotic from leptotene and became aligned by their centromeres at pachytene (Fig. 6N). Positive heteropycnosis was obvious during metaphase I (Fig. 6O), but it became less intensive during metaphase II (Fig. 6P). These results correspond to the information available for the only trochanteriid analysed to date, *Plator pandeae* Tikader, 1969 from India, which also exhibits 2n♂ = 22, X_1_X_2_0 (Srivastava and Shukla 1986).

### IVc. Dionycha part B

Following the results of Wheeler et al. (2016), this group forms a monophyletic clade comprising eight families with mostly unresolved relationships. In the present study, we analysed three of them.

**Cheiracanthiidae Wagner, 1887**

**Figure 7.**
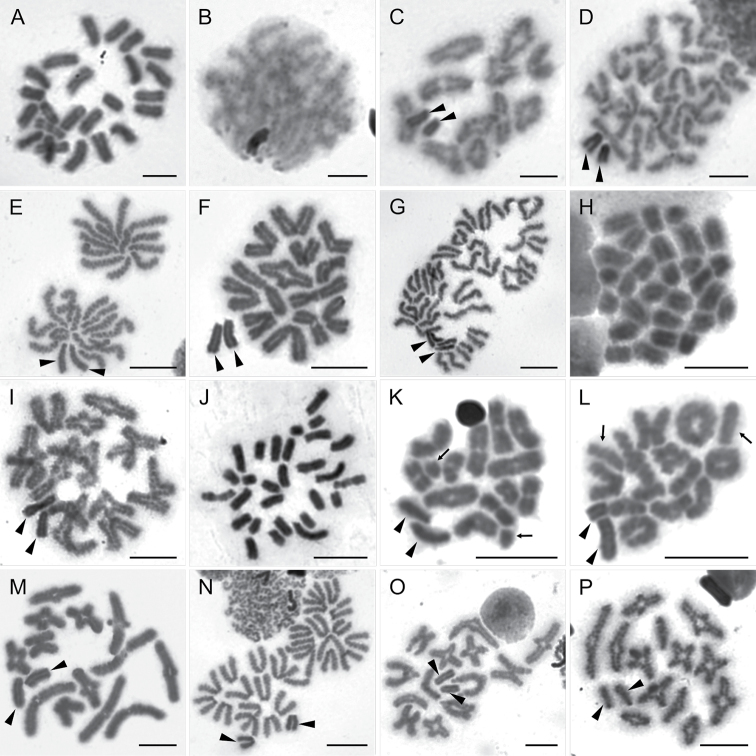
Chromosomes of Cheiracanthiidae (**A–D)** and Salticidae (**E–P**). *Cheiramiona kirkspriggsi* (2n♂ = 24, X_1_X_2_0) **A** mitotic metaphase **B** pachytene, sex chromosomes associate on the periphery of nucleus **C** diakinesis with positively heteropycnotic X_1_X_2_**D** metaphase II **E***Baryphas ahenus*, (2n♂ = 28, X_1_X_2_0) prometaphase II. *Cyrba lineata* (2n♂ = 28, X_1_X_2_0) **F** diakinesis **G** metaphase II. *Holcolaetis zuluensis* (2n♂ = 28, X_1_X_2_0) **H** mitotic metaphase **I** diakinesis, with positively heteropycnotic X_1_X_2_. *Myrmarachne laurentina* (2n♂ = 28, X_1_X_2_0) **J** mitotic metaphase **K** early metaphase I, note one bivalent with early segregation (arrows) **L***Menemerus minshullae* (2n♂ = 28, X_1_X_2_0) diakinesis, note one bivalent with early segregation (arrows) **M** Diakinesis of *Nigorella hirsuta* (2n♂ = 28, X_1_X_2_0) **N** Metaphase II of *Thyene ogdeni* (2n♂ = 28, X_1_X_2_0) X_1_X_2_ are positively heteropycnotic **O***Thyenula haddadi* (2n♂ = 28, X_1_X_2_0) diakinesis **P***Thyenula leighi* (2n♂ = 28, X_1_X_2_0) diakinesis. Arrowheads indicate sex chromosomes. Scale bars: 5 μm (**A–D**), 10 μm (**E–P**).

The family, restored by Ono and Ogata (2018), currently includes 12 genera and more than 350 species (World Spider Catalog 2019). In *Cheiramiona kirkspriggsi* Lotz, 2015, we identified 2n♂ = 24, X_1_X_2_0 (Fig. 7A, D). All autosomes were acrocentrics and gradually decreased in length. The X_1_ and X_2_ differed in length substantially (the ratio of sex chromosomes – 1:0.77) during diakinesis (Fig. 7C). They also showed positive heteropycnosis during the whole course of meiosis (we did not observe anaphase II). Gonosomes started associating at zygotene by their (probably distal) ends, and were arranged in parallel during pachytene and diakinesis (Fig. 7B, C). They were localized close together during meiosis II too (Fig. 7D).

The genus *Cheiracanthium* Koch, 1839, closely related to *Cheiramiona* Lotz & Dippenaar-Schoeman, 1999 (Lotz and Dippenaar-Schoeman 1999, Ramírez 2014), representing the most cytogenetically examined cheiracanthiid genus so far (Araujo et al. 2019), commonly displays 2n♂ = 26 and X_1_X_2_0 (see Araujo et al. 2019). The karyotype of *Cheiramiona kirkspriggsi* was probably derived from this state, by tandem fusion or a series of lesser translocations, leading to degeneration of the donor autosome, as the reduction of 2n is presumably the leading trend of karyotype evolution in Entelegynae (Kořínková and Král 2013). The X_1_X_2_0 sex chromosome system is also present in most analysed cheiracanthiid species (see Araujo et al. 2019), with exception of *Cheiracanthium saraswatii* Tikader, 1962, *C. melanostomum* (Thorell, 1895) and *C. murinum* (Thorell, 1895), with X_1_X_2_X_3_0 (Datta and Chatterjee 1983, Srivastava and Shukla 1986). Interestingly, *C. saraswatii* and *C. melanostomum* also possess a distinctly higher number of chromosomes (2n♂ = 43), while the rest of the species have 2n♂ = 22–28, with 26 being the most frequent.

**Salticidae Blackwall, 1841**

Jumping spiders are the most diverse spider family, with about more than 6100 species globally and 350 species distributed in South Africa (Wesołowska and Haddad 2018, World Spider Catalog 2019). Paralleling their diversity, with 160 karyotyped species the family is also well-investigated in terms of cytogenetics. Most of the species exhibit 2n♂ = 28, X_1_X_2_0 (see Araujo et al. 2019). However, numerous cases of neo sex chromosome formation have been reported in American representatives (Maddison and Leduc-Robert 2013).

We analysed nine species of South African salticids. Consistent with the majority of published data, we found a 2n♂ = 28, X_1_X_2_0 system in all of the species analysed in this study, namely *Baryphas ahenus* Simon, 1902 (Fig. 7E), *Cyrba lineata* Wanless, 1984 (Fig. 7F, G), *Holcolaetis zuluensis* Lawrence, 1937 (Fig. 7H, I), *Myrmarachne laurentina* Bacelar, 1953 (Fig. 7J, K), *Menemerus minshullae* Wesołowska, 1999 (Fig. 7L, 9E), *Nigorella hirsuta* Wesołowska, 2009 (Fig. 7M), *Thyene ogdeni* Peckham & Peckham, 1903 (Fig. 7N) and two species of *Thyenula* Simon, 1902, *T. haddadi* Wesołowska, Azarkina & Russell-Smith, 2014 (Fig. 7O) and *T. leighi* (Peckham & Peckham, 1903) (Fig. 7P). A completely acrocentric karyotype was detected in *B. ahenus* (Fig. 7E), *C. lineata* (Fig. 7G), *M. minshullae* (Fig. 9F), *N. hirsuta* (Fig. 9G), *T. ogdeni* (Fig. 7N) and both species of *Thyenula* (Fig. 9H, I). Unfortunately, in the remaining two species the chromosome plates were of insufficient quality to allow the identification of the morphology of the whole chromosome complement. Our dataset thus further supports the conservatism of 2n in this highly diversified group of spiders (Araujo et al. 2019).

**Selenopidae Simon, 1897**

**Figure 8.**
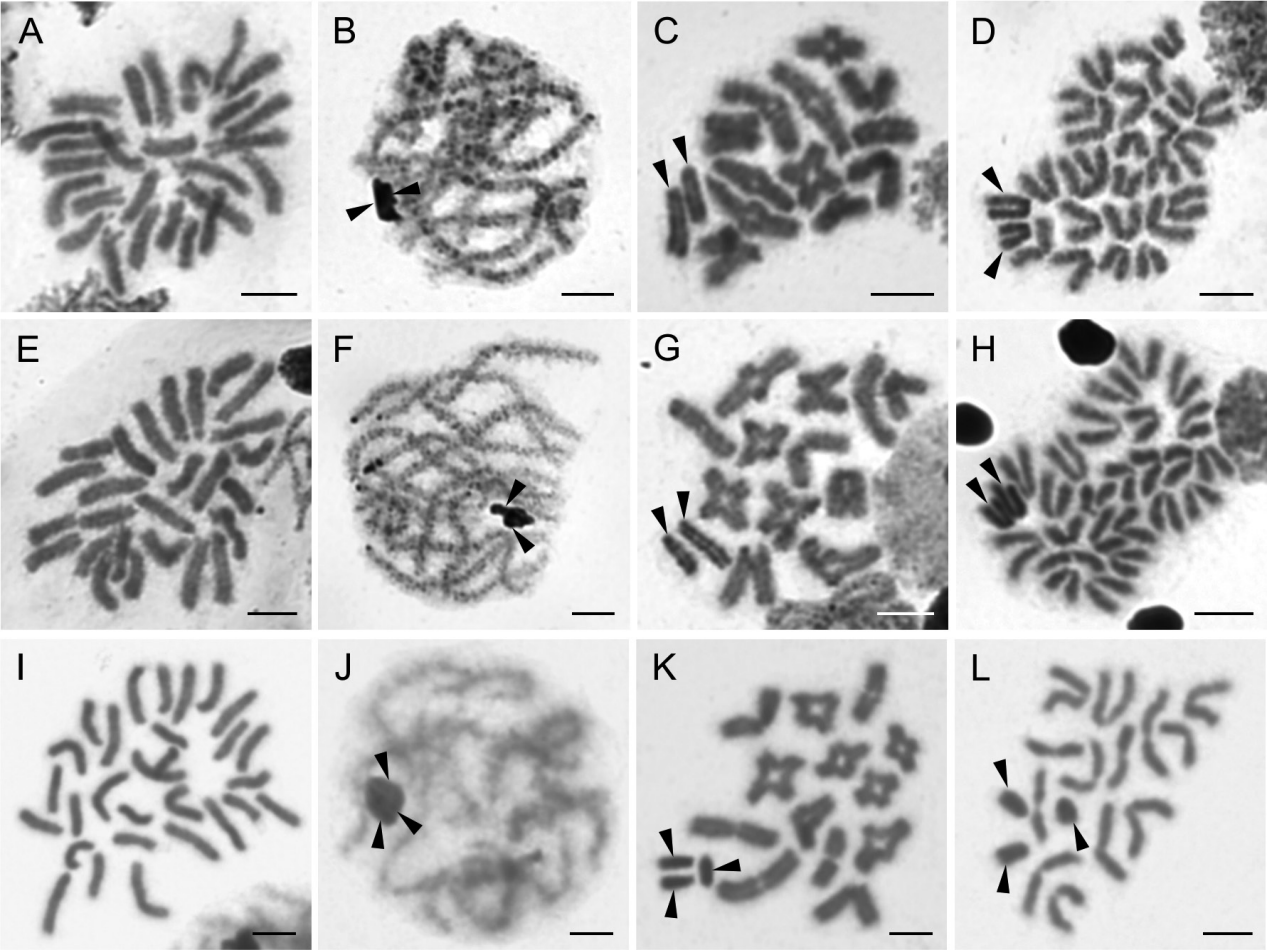
Chromosomes of Selenopidae. *Anyphops* sp. (Mossel Bay) (2n♂ = 26, X_1_X_2_0) **A** mitotic metaphase **B** pachytene, note close association of sex chromosomes **C** diakinesis **D** metaphase II, note positive heteropycnosis of X chromosomes. *Selenops* sp. 1 (Ophathe) (2n♂ = 26, X_1_X_2_0) **E** mitotic metaphase **F** pachytene, note close association of X_1_X_2_**G** diakinesis **H** metaphase II. *Selenops* sp. 2 (Namibia) (2n♂ = 29, X_1_X_2_X_3_0) **I** mitotic metaphase **J** pachytene, note close association of X_1_X_2_X_3_**K** metaphase I **L** half of metaphase II with sex chromosomes. Arrowheads indicate sex chromosomes. Scale bars: 5 μm.

Selenopids can be considered a smaller family, distributed in the tropics and subtropics (Jocqué and Dippenaar-Schoeman 2006). We analysed two specimens of *Anyphops* Benoit, 1968 and two subadult males of *Selenops* Latreille, 1819 from different localities in South Africa, and one male from Namibia (Table 1). Specimens of both genera from South Africa displayed a similar karyotype of 2n♂ = 26 (Fig. 8A, E), with acrocentric chromosomes gradually decreasing in length (Fig. 8A,E, D, H). The sex chromosome system X_1_X_2_0 was identified in all South African specimens; the X_1_ and X_2_ differed in length (the ratio of sex chromosomes – 1:0.75–0.78) (Fig. 8C, G). Both sex chromosomes showed intensive positive heteropycnosis and parallel association during pachytene (Fig. 8B, F). Their pairing, location on the periphery of the nucleus and positive heteropycnosis also persisted during diakinesis (Fig. 8C, G) and metaphase II (Fig. 8D, H). On the other hand, the male of *Selenops* sp. 2 from Namibia displayed 2n = 29 (Fig. 8I), with acrocentric morphology of all chromosomes (Fig. 8I, L) and an X_1_X_2_X_3_0 sex chromosome system (Fig. 8K). In contrast to the species from South Africa, the sex chromosomes of the Namibian representative were of similar length (Table 1), but the characteristics concerning the heteropycnosis and behaviour during meiosis were similar to the other species. The sex chromosomes were associated during pachytene (Fig. 8J) and positively heteropycnotic during the whole meiosis (Fig. 8J–L).

**Figure 9.**
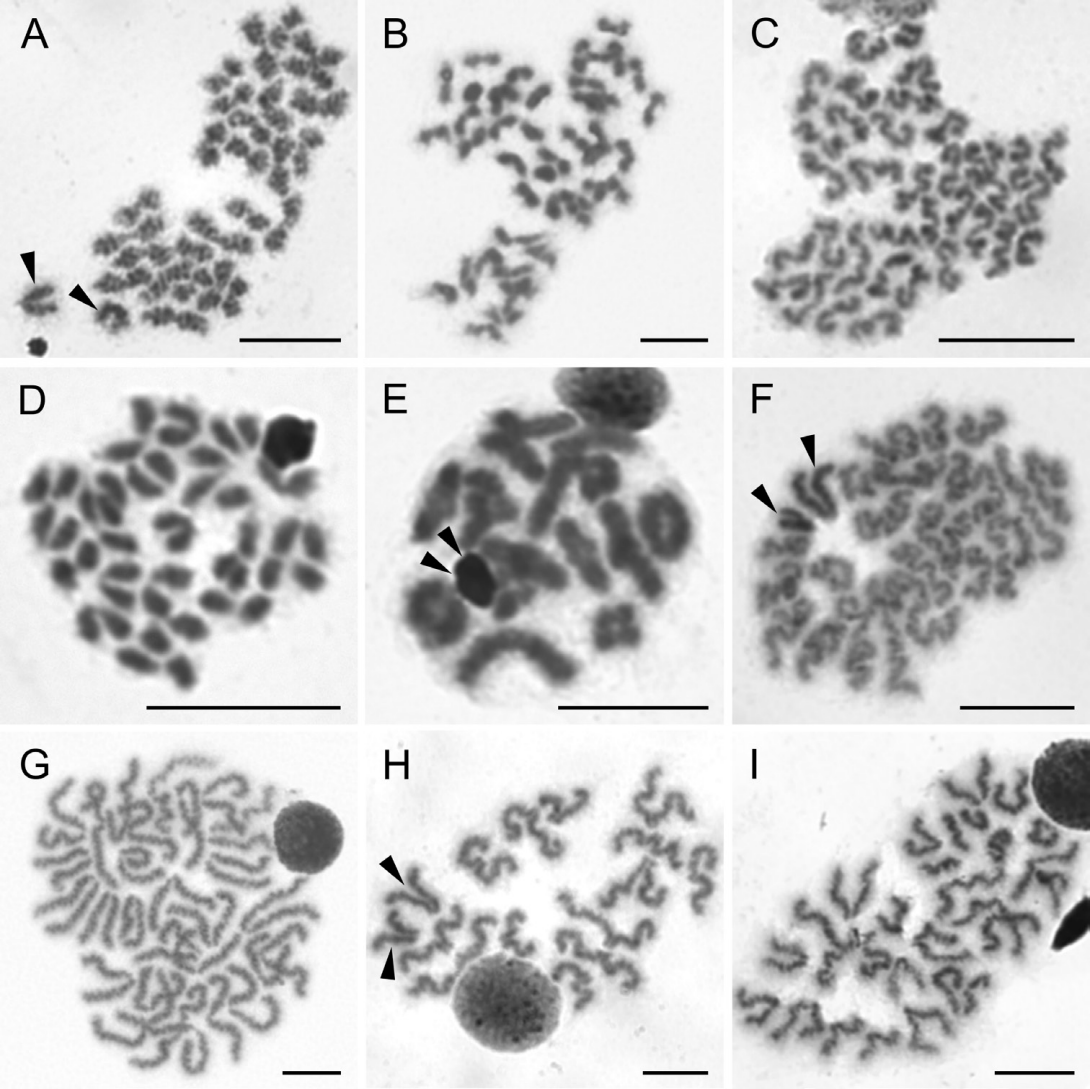
Chromosomes of Ctenidae (**A**), Ammoxenidae (**B**), Gnaphosidae (**C, D**), and Salticidae (**E–I**). Ctenus cf. pulchriventris (2n♂ = 28, X_1_X_2_0) **A** prometaphase II. *Ammoxenus amphalodes* (2n♂ = 22, X_1_X_2_0) **B** two metaphases II. *Camillina maun* (2n♂ = 22, X_1_X_2_0) **C** metaphase II. *Zelotes* sp. (2n♂ = 22, X_1_X_2_0) **D** metaphase II. *Menemerus minshullae* (2n♂ = 28, X_1_X_2_0) **E** metaphase I **F** metaphase II. *Nigorella hirsuta* (2n♂ = 28, X_1_X_2_0) **G** metaphase II. *Thyenula haddadi* (2n♂ = 28, X_1_X_2_0) **H** metaphase II, one sister cell with sex chromosomes. *Thyenula leighi* (2n♂ = 28, X_1_X_2_0) **I** diakinesis. Arrowheads indicate sex chromosomes. Scale bars: 10 μm.

Interestingly, only the data obtained from the Namibian specimen, namely the 2n and the sex chromosome system, were comparable to karyotypes described in three other karyotyped selenopids (Suzuki 1952). The 2n♂ = 29, X_1_X_2_X_3_0 was reported from species belonging to the genera *Makdiops* Crews & Harvey, 2011 and *Selenops* from India (Sharma et al. 1959, Mittal 1966, Prakash and Prakash 2014). This indicates that the reduction of 2n could have occurred in southern Africa. Interestingly, species with the reduced autosome number also possess an X_1_X_2_0 system, which could indicate that the ancestral sex chromosome constitution was X_1_X_2_X_3_, with a subsequent reduction to X_1_X_2_0. However, additional research on this topic will be necessary in order to answer this question.

### Distribution of major rDNA loci

**Figure 10.**
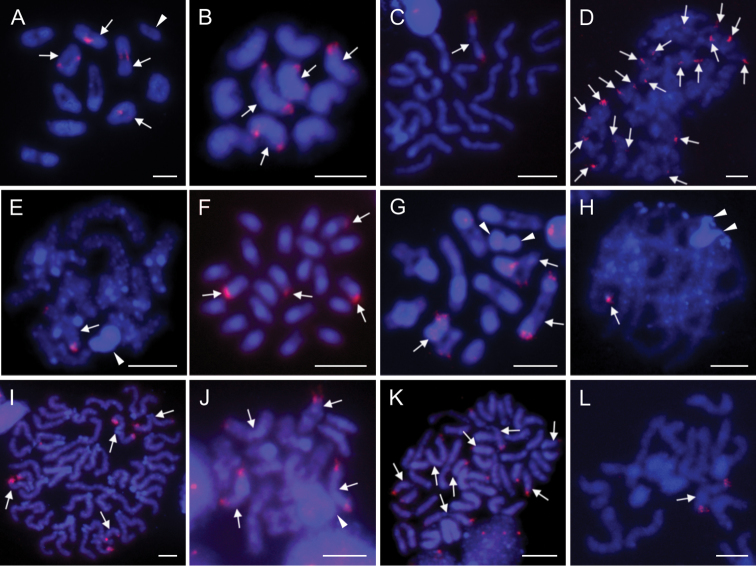
Chromosomes of entelegyne spiders from South Africa after FISH with 18S rDNA probe (red signal). Arrows point on bivalents with signals (**A, E, G, H, J**), certain chromosomes with signals (**B, C, F, I, K, L**), or certain signals (**D**) **A**Argyrodes cf. convivans (Theridiidae), diplotene **B**Argyrodes cf. convivans, half of metaphase II (without sex chromosomes), note distal (opposite centromere) positions of loci **C***Olios* sp. from South Africa (Sparassidae), metaphase II, one sister cell without sex chromosomes, note distal position of locus **D***Olios* sp. from Namibia (Sparassidae), metaphase II, signals at distal parts of acrocentric chromosomes **E***Afroceto plana* (Trachelidae), diplotene **F***Zelotes sclateri* (Gnaphosidae), mitotic metaphase **G***Platyoides walteri* (Trochanteriidae), diplotene **H***Cheiramiona kirkspriggsi* (Cheiracanthiidae), pachytene, with distal signal on one bivalent **I***Thyenula haddadi* (Salticidae), metaphase II, distal signals on two chromosome pairs **J***Anyphops* sp. (Mossel Bay) (Selenopidae), diplotene **K***Selenops* sp. 1 (Pongola Reserve) (2n♂ = 26) (Selenopidae), metaphase II, two sister cells **L***Selenops* sp. 2 (Selenopidae), half of metaphase II. Arrowheads indicate sex chromosomes, where distinguished. Scale bars: 5 μm.

We applied 18S rDNA FISH on 11 species from eight different families, including: i) one araneoid (Fig. 10A, B); ii) two species belonging to the non-dionychan RTA clade (family Sparassidae, Fig. 10C, D); iii) three gnaphosoids (Fig. 10E–G); and iv) five non-gnaphosoid Dionycha (Fig. 10H–L), including three members of the family Selenopidae. Numbers of clusters varied from one to five loci (Table 1). We did not observe an 18S rDNA signal on the X chromosome in any of the analysed species. Most of the species possessed a single locus, namely *Olios* sp. from South Africa (Sparassidae) (Fig. 10C), *Afroceto plana* (Trachelidae) (Fig. 10E), *Cheiramiona kirkspriggsi* (Cheiracanthiidae) (Fig. 10H), and *Selenops* sp. 2 from Namibia (Selenopidae) (Fig. 10L). The signals were located in the distal positions on the long arms in all species (e.g. Fig. 10C, L). Moreover, in less spiralized chromosomes (e.g. in pachytene), the signal could be confirmed in a terminal position (Fig. 10H). We found two distal loci in *Zelotes sclateri* (Gnaphosidae) (Fig. 10F) and *Thyenula haddadi* (Salticidae) (Fig. 10I). Interestingly, we identified three 18S rDNA loci in *Platyoides walteri* (Fig. 10G), and even four in Argyrodes cf. convivans (Fig. 10A, B), all of them in distal positions.

In some cases, we found a different number of loci in members of the same family. Namely, two South African representatives of Selenopidae (*Selenops* sp. 1 Ophathe/Pongola and *Anyphops* sp. Ndumo/Mossel Bay) displayed four distal loci (Fig. 10J, K), while *Selenops* sp. 2 from Namibia only had one (Fig. 10L). We observed even higher variation between the two analysed species from the family Sparassidae. One male from South Africa only had a single 18S rDNA locus (Fig. 10C), whereas we found five loci in *Olios* sp. from Namibia (Fig. 10D), all of them likely in a distal position. Both sparassids differed in the number of clusters, despite having the same 2n. This indicates that genome dynamics in entelegynes could be substantial, even though the genome rearrangements do not manifest themselves by changing of 2n and chromosome morphology.

The position of major rDNA loci has previously been examined in a limited number of entelegynes, namely in one lycosid species (Forman et al. 2013) and four ctenids (Rincão et al. 2017), but we were able to observe broad variation in their number, ranging from one (this study) up to ten (Forman et al. 2013). Notably, the number of loci can differ at family level (this study), or even intraspecifically (Forman et al. 2013). The number of major rDNA loci in entelegynes is very dynamic and more work is necessary to evaluate its usefulness for the cyto-systematics of spiders. Interestingly, the position of the loci on the distal ends of the acrocentric chromosomes seems to be conservative in Entelegynae. The absence of major rDNA loci on the X chromosomes in entelegynes contrasts with our knowledge generated from the basal groups of haplogyne spiders, where the NORs have been found on sex chromosomes via silver impregnation (Král et al. 2006).

### General trends of entelegyne karyotype evolution

Our cytogenetic results from southern African entelegynes fit with our knowledge of the general trends in karyotype diversification of the group. Compared to other major clades of spiders (Král et al. 2006, 2013, 2019), Entelegynae karyotypes are more homogenous in 2n ranges and very conservative in morphology, which is nearly exclusively acrocentric (Araujo et al. 2019, this study). The X_1_X_2_0 sex determination system is dominant in entelegynes. Alternatively, the presence of X0 and X_1_X_2_X_3_0 is also common (Araujo et al. 2013, Kořínková and Král 2013, this study). The leading trend of karyotype diversification is a decrease in diploid counts, which convergently occurred among the groups.

In case of Araneoidea, our results from *Pachygnatha* and *Theridion* showed the typical karyotype conservatism in both Tetragnathidae and Theridiidae. On the other hand, *Argyrodes* (Theridiidae) displayed chromosomal rearrangements unusual for entelegynes. Stávale et al. (2010) described inversions of autosomes in *A. elevatus*, accompanied by a Robertsonian fusion of X chromosomes, leading to an X0 system. Because we can confirm acrocentric morphology of the sex chromosomes in both *Argyrodes* species examined here, it is thus likely that the X0 sex system evolved via tandem fusion of an ancestral acrocentric X_1_ and X_2_. An alternate scenario for the origin of the X0 sex system in South African *Argyrodes* could be explained by centric fusion of X chromosomes and their subsequent pericentric inversion. In both cases, the X0 sex chromosome systems among the *Argyrodes* species evolved independently. This makes *Argyrodes* an interesting model for research of X_1_X_2_0/X0 transition mechanisms.

In comparison to other Entelegynae families, Oecobioidea karyotypes represented a dynamic system. Hersiliidae, despite limited data availability for only four species (Bole-Gowda 1958, Kumbıçak et al. 2018, this study), displayed three different diploid counts. Interestingly, Oecobiidae showed the highest diversity in 2n among Entelegynae families, approaching both upper and lower ranges of Entelegynae diploid number (or fundamental number, respectively), ranging from 2n♂ = 19, X0 (this study) to 2n♂ = 42, X_1_X_2_0 (Suzuki 1950). Therefore, the Oecobioidea present an intriguing group that could provide important insights into some fundamental trends of Entelegynae karyotype evolution. Further studies of the African fauna, notably, endemic genera of Hersiliidae (*Tyrotama* Foord & Dippenaar-Schoeman, 2005) and Oecobiidae (*Uroecobius* Kullmann & Zimmermann, 1976 and *Urocteana* Roewer, 1961) could contribute significantly to this topic in the future.

Despite the rare utilization of cytogenetic markers in Entelegynae phylogenetics, the results presented in this paper could have an implication for the group’s systematics. The RTA clade represents the most diversified group of entelegynes. The non-dionychan members of the RTA clade analysed in this paper showed a broad range of diploid numbers in entelegynes with acrocentric chromosomes. The 2n of examined sparassids ranked among the highest in entelegynes, neighbouring the proposed ancestral state for the group (2n♂ = 42, Král et al. 2006). We also described karyotypes of two members of the families Ctenidae and Oxyopidae, traditionally placed within the superfamily Lycosoidea. Both species analysed showed 2n♂ = 28, X_1_X_2_0 in males, which is a hypothesised ancestral condition for whole Lycosoidea (Dolejš et al. 2011). In both families, a decreasing autosome number has been reported (Stávale et al. 2011, Araujo et al. 2014), with exception of some Ctenidae representatives, where chromosome count increases due to the formation of a X_1_X_2_X_3_0 system (e.g. Araujo et al. 2014). Finding the same condition in basal Thomisidae (*Borboropactus*) could mean that 2n♂ = 28 is an ancestral state for the whole family, which also fits with the proposed placement of thomisids within the Lycosoidea (Polotow et al. 2015), which was also supported by phylogenomic analyses (Fernández et al. 2018). However, the karyotype formula 2n♂ = 28, X_1_X_2_0 is common among the Entelegynae families, and it is also proposed as the ancestral condition for many of them (Kořínková and Král 2013). Therefore, the diploid number itself cannot be interpreted as a strong argument for Thomisidae placement within the Lycosoidea.

All representatives of the superfamily Gnaphosoidea analysed in this paper displayed the same karyotype of 22 acrocentric chromosomes and X_1_X_2_0, which confirmed that such a constitution is widespread not only within Gnaphosidae, but also among the other Gnaphosoidea families. Despite the limited data, higher 2n has not been found in any species of Gnaphosidae, Ammoxenidae, Gallieniellidae and Trochanteriidae (see Araujo et al. 2019, this study). These findings suggest that Gnaphosoidea are extremely conservative, and the few species that exhibit a formula other than 22 acrocentrics represent an exception to the rule. For example, Trachelidae, a group closely related to the Gnaphosoidea, comprises species with both 22 and 24 chromosomes (Suzuki 1952, Datta and Chatterjee 1983). The 2n♂ = 22, X_1_X_2_0 can be thus considered an ancestral state for both Gnaphosidae, as well as the majority of Gnaphosoidea families. The karyotypes of subfamily Prodidominae are in contrast with the characteristics of Gnaphosoidea listed above. We detected three more autosome pairs (or two if we will include *Trachelas* sp.) in their karyotypes. An increasing autosomal number is not common in entelegynes (Suzuki 1954), and fissions of acrocentric chromosomes in general are very unlikely. If we consider prodidomines as an internal group of Gnaphosidae, we would have to assume three such independent events. For this reason, we can safely conclude that prodidomines’ position within Gnaphosidae family is highly unlikely. Our conclusion is further supported by the results of the molecular analyses (Wheeler et al. 2016) that recovered Prodidomidae as a sister clade to Dionycha.

Our results of the Dionycha part B clade taxa confirm the 2n conservatism within the families Salticidae and Cheiracanthiidae. On the other hand, a substantial variability was observed in Selenopidae. Both *Selenops* and *Anyphops* individuals from South Africa share karyotype features, namely: 2n♂ = 26, X_1_X_2_0 and four 18S rDNA loci, while *Selenops* sp. 2 from Namibia possesses 2n♂ = 29, X_1_X_2_X_3_0 and a single 18S rDNA locus.

## Conclusions

This study improves our knowledge about entelegyne karyotypes and brings new information about taxa from an understudied biogeographical region. The data proceeding from South Africa and Namibia are consistent with the information available for entelegyne karyotypes from other continents. Here, we confirmed the stability of the karyotype characteristics, namely acrocentric morphology, the prevalence of X_1_X_2_0, and relatively small ranges of 2n in most families. On the other hand, we found variability of 2n within the families Hersiliidae, Oecobiidae, Gnaphosidae, Selenopidae and Thomisidae. Our cytogenetic data challenge the current placement of Prodidomidae as an internal group of Gnaphosidae, although admittedly further taxa should be analysed to resolve this conundrum, and thus highlight the utility of cytogenetics for taxonomy and systematics. Our study expands our knowledge about major rDNA loci distribution in the Entelegynae, and reveals surprising variability in the number of loci among certain taxa.
